# Supplementary material for: Enhancing Maize Transformation and Targeted Mutagenesis through the Assistance of Non-Integrating Wus2 Vector
Source: Plants (Basel). 2023 Jul 28;12(15):2799. doi: 10.3390/plants12152799 (PMC10420852; doi:10.3390/plants12152799)
Supplement: Supplementary file 1 [file plants-12-02799-s001.zip › plants-2475409-supplementary.pdf]

Supplement Materials

Enhancing Maize Transformation and Targeted Mutagenesis Through the Assistance of Non-Integrating Wus2 Vector

Minjeong Kang[1,2,3], Keunsub Lee[1,2], Qing Ji[2], Sehiza Grosic[2], and Kan Wang[1,2]*

[1]Department of Agronomy, Iowa State University, Ames, Iowa, USA

[2]Crop Bioengineering Center, Iowa State University, Ames, Iowa, USA

[3]Interdepartmental Plant Biology Major, Iowa State University, Ames, Iowa, USA

*Author for correspondence: kanwang@iastate.edu

Keywords: *Wuschel2*, morphogenic transcription factor, *Agrobacterium*-mediated transformation, ternary vector system, CRISPR-Cas9, transgene-free editing

Figure S1. Transgene-free edited B73 plants confirmed by PCR.

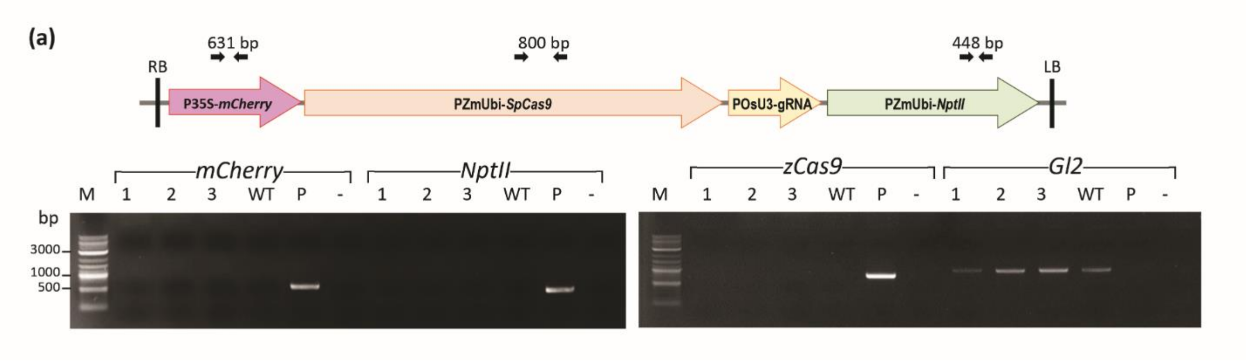

Figure S1. Transgene-free edited B73 plants confirmed by PCR. Three transgene-free edited plants (B73-R1~R3) were tested for the presence of three genes encoded on the GOI T-DNA: mCherry, NptII, and zCas9. Gl2, glossy2; WT, wildtype control; P, pKL2359 plasmid DNA as a positive control; -, water control for PCR.

Table S1. Summary of *Glossy2* genotyping analysis in B104.

	**Homozygous**	**Biallelic**	**Heterozygous**	**Mosaic**	**Wildtype**	**Total**
Control	1 (5.3%)	6 (31.6%)	0 (0%)	10 (52.6%)	2 (10.5%)	19 (100%)
LBA4404Thy-	8 (22.9%)	13 (37.1%)	0 (0%)	8 (22.9%)	6 (17.1%)	35 (100%)
EHA105Thy-	3 (21.4%)	2 (14.3%)	0 (0%)	7 (50%)	2 (14.3%)	14 (100%)
EHA105TR	5 (19.2%)	10 (38.5%)	2 (7.7%)	4 (15.4%)	5 (19.2%)	26 (100%)

Homozygous, one mutant sequence without wild type; Biallelic, two different mutant sequences; Heterozygous, wild type sequence and one mutant sequence; Mosaic, three or more mutant sequences in a single plant sample. LBA4404Thy-, auxotrophic LBA4404 strain with *thymidine synthase* gene (*thyA*) knock-out; EHA105Thy-, auxotrophic EHA105 strain with *thyA* knock-out; EHA105TR, *recA*-deficient auxotrophic EHA105Thy- strain.

Table S2. List of primers used in this study

Primer name	Description	Sequence (5'-3')
ZmWUS2-F1	PCR primer to amplify maize Wus2 and TIn2-1 terminator cassette from PHP97334 [1]	TTTAACTTAGCCTAGGATCCCATATTCACTCCCATGGCGG
TIN2-1-R1	PCR primer to amplify maize Wus2 and TIn2-1 terminator cassette from PHP97334 [1]	AACTGTCGGTCCAATAGACGCCGCTCTCTCTCTCCTTGCTA
3xENH-F1	PCR primer to amplify 3x viral enhancers and maize Ubiquitin promoter from PHP97334 [1]	AGTCGACCTGCAGGCATGCAAATCGACCGAAGCTTGCATG
PZmUbi-R1	PCR primer to amplify 3x viral enhancers and maize Ubiquitin promoter from PHP97334 [1]	GGATCCTAGGCTAAGTTAAAGTC
P35-RUBY-F1	PCR primer to amplify the RUBY cassette from pCBL101-RUBY [2]	TGATTACGAATTCGAGCTCGACACTGATAGTTTTGAGACTTT
P35-RUBY-R1	PCR primer to amplify the RUBY cassette from pCBL101-RUBY [2]	CGACTCTAGAGGATCCCCGGAAAACAGTTTTCCCAATGCCA
DODA-R1	PCR primer to amplify the RUBY cassette from pCBL101-RUBY [2]	CGAGATTGTAGTGGTAGGTG
DODA-F1	PCR primer to amplify the RUBY cassette from pCBL101-RUBY [2]	GACGGCACCTACCACTACAA
zCas9-F	PCR primer to check the presence of zCas9 [3]	CCGATTCTGGAGAAGATGGA
zCas9-R	PCR primer to check the presence of zCas9 [3]	TCGAAGAGATGGGCGTAAGT
zCas9-seq-F2	PCR primer to check the presence of zCas9	CCTGTTCGGGAATCTCATTG
zCas9-seq-R2	PCR primer to check the presence of zCas9	ACTCGTACAGGAGCGAGTGC
zCas9-seq-F3	PCR primer to check the presence of zCas9	GTATGTGACCGAGGGCATGA
zCas9-seq-R3	PCR primer to check the presence of zCas9	GGGTGCTCCTTGAGGATCTG
zCas9-seq-F4	PCR primer to check the presence of zCas9	CAGCTGCAGAATGAGAAGCTC
zCas9-seq-R4	PCR primer to check the presence of zCas9	AAATCCCTGCCCTTGTCC
zCas9-seq-F5	PCR primer to check the presence of zCas9	ATTCTGCCTAAGCGGAACAG
zCas9-seq-R5	PCR primer to check the presence of zCas9	GTGTGCGCAATGAAACTGAT
Zm-gl2-F2	PCR primer to amplify maize Glossy2 for genotyping analysis [3]	CACAGCCTTGCAATCAATTC
Zm-gl2-R2	PCR primer to amplify maize Glossy2 for genotyping analysis [3]	GCTGACGTGGAAGGAGTAGC
ZmGl2-exon2-F1	Sequencing primer for Glossy2 fragment for genotyping analysis [3]	ACACCGTGTCTTCGTCAAAA
Ruby_F1	PCR primer to check the presence of RUBY from the transgenic plants	TTTACGACCAGCCTCAACCT
Ruby_R1	PCR primer to check the presence of RUBY from the transgenic plants	GCAGGTTGATGATGTCGGAC
mCherry-F1	PCR primer to check the presence of mCherry from the transgenic plants	GGGCGAGGAGGATAACATGG
mCherry-R1	PCR primer to check the presence of mCherry from the transgenic plants	GGTGTAGTCCTCGTTGTGGG
mCherry-F2	PCR primer to check the presence of mCherry from the transgenic plants	TTCAAGGTGCACATGGAGGG
mCherry-R2	PCR primer to check the presence of mCherry from the transgenic plants	GATGTTGACGTTGTAGGCGC
NptII_F1	PCR primer to check the presence of NptII from the transgenic plants	GAATGAACTGCAGGACGAGG
NptII_R1	PCR primer to check the presence of NptII from the transgenic plants	GAATCCAGAAAAGCGGCCAT

[1] Wang, N., Ryan, L., Sardesai, N., Wu, E., Lenderts, B., Lowe, K., Che, P., Anand, A., Worden, A., van Dyk, D. and Barone, P., 2023. Leaf transformation for efficient random integration and targeted genome modification in maize and sorghum. Nature Plants, 9(2), pp.255-270.

[2] Lee, K., Kang, M., Ji, Q., Grosic, S. and Wang, K., 2023. New T-DNA binary vectors with NptII selection and RUBY reporter for efficient maize transformation and targeted mutagenesis. Plant Physiology, p.kiad231.

[3] Lee, K., Zhang, Y., Kleinstiver, B.P., Guo, J.A., Aryee, M.J., Miller, J., Malzahn, A., Zarecor, S., Lawrence-Dill, C.J., Joung, J.K. and Qi, Y., 2019. Activities and specificities of CRISPR/Cas9 and Cas12a nucleases for targeted mutagenesis in maize. Plant biotechnology journal, 17(2), pp.362-372.

Table S3. List of transgenic B73 plants with indel mutations.

Plant ID	T0 genotype		*Glossy2* sequence	Indel mutation	Contribution%
	**WT**	Allele 1:	**TTGGTCACAGATCACAAACTTCAAATGCGGTGGGCTGGCGCTGGGGTTCAGCT**	0 bp	
		Allele 2:	**TTGGTCACAGATCACAAACTTCAAATGCGGTGGGCTGGCGCTGGGGTTCAGCT**	0 bp	
**B4-A-15**	**BI**	Allele 1:	**TTGGTCACAGATCACAAACTTCAAAATGCGGTGGGCTGGCGCTGGGGTTCAGCT**	+1 bp	45
		Allele 2:	**TTGGTCACAGATCACAAACTTCA----------GCTGGCGCTGGGGTTCAGCT**	-10 bp	41
**B5-A-06A**	**BI**	Allele 1:	**TTGGTCACAGATCACAAACTT---ATGCGGTGGGCTGGCGCTGGGGTTCAGCT**	-3 bp	89
		Allele 2:	**TTGGTCACAGATCACAAA-----------------TGGCGCTGGGGTTCAGCT**	-17 bp	1
**B5-A-16A**	**HT**	Allele 1:	**TTGGTCACAGATCACAAACTT---ATGCGGTGGGCTGGCGCTGGGGTTCAGCT**	-3 bp	64
		Allele 2:	**TTGGTCACAGATCACAAACTTCAAATGCGGTGGGCTGGCGCTGGGGTTCAGCT**	0 bp	26
**B4-A-11**	**MO**	Allele 1:	**TTGGTCACAGATCACAAACTTCAAATGCGGTGGGCTGGCGCTGGGGTTCAGCT**	0 bp	12
		Allele 2:	**TTGGTCACAGATCACAAAC---------------CTGGCGCTGGGGTTCAGCT**	-15 bp	7
		Allele 3:	**TTGGTCACAGATCACAAACTTCAA--------------CGCTGGGGTTCAGCT**	-14 bp	7
**B5-A-01D**	**MO**	Allele 1:	**TTGGTCACAGATCACAAACTTCAAATGCGGTGGGCTGGCGCTGGGGTTCAGCT**	0 bp	73
		Allele 2:	**TTGGTCACAGATCACAAACTT---------------GGCGCTGGGGTTCAGCT**	-15 bp	14
		Allele 3:	**TTGGTCACAGATCACAAACTTCAANATGCGGTGGGCTGGCGCTGGGGTTCAGCT**	+1 bp	6
**B5-A-03A**	**MO**	Allele 1:	**TTGGTCACAGATCACAAACTTCAANATGCGGTGGGCTGGCGCTGGGGTTCAGCT**	+1 bp	30
		Allele 2:	**TTGGTCACAGATCACAAACT----ATGCGGTGGGCTGGCGCTGGGGTTCAGCT**	-4 bp	15
		Allele 3:	**TTGGTCACAGATCACAAACTTCAA----GGTGGGCTGGCGCTGGGGTTCAGCT**	-4 bp	7
**B5-A-11A**	**MO**	Allele 1:	**TTGGTCACAGATCACAAACTTCAAATGCGGTGGGCTGGCGCTGGGGTTCAGCT**	0 bp	62
		Allele 2:	**TTGGTCACAGATCACAAACTTC---TGCGGTGGGCTGGCGCTGGGGTTCAGCT**	-3 bp	16
		Allele 3:	**TTGGTCACAGA---------------------------------GGTTCAGCT**	-33 bp	10

WT, wild type; HT, heterozygous mutation; BI, biallelic mutation; MO, mosaic mutation showing more than 3 mutations at the target site. PAM (blue) and protospacer (red) sequences are highlighted. Inserted bases (A, N) are underlined. Contribution % represents relative proportions of indel sequence ($p < 0.001$) in each sample.
